# The Medical Society of the County of Erie

**Published:** 1891-07

**Authors:** 


### THE MEDICAL SOCIETY OF THE COUNTY OF ERIE.

IT will be observed, by reference to another column, that this Society held an important meeting in the early days of June. The matters of greatest interest grouped around the report of the Board of Censors, which led to the expulsion of one of the members, to the acquittal of one, and to receiving charges in another case.

The JOURNAL regrets that any member of the profession should bring himself within the range of censure in the conduct of his professional duties, but it is difficult to see how the Society could have disposed of this delicate business in a more just and generous way than it did. The time appears to have arrived when young men must be taught that they assume some other obligations than merely the earning of their maintenance when they enter the ranks of medicine; so, too, must men coming into our land from foreign shores, because it will not answer for them to act on the principle that a man has a right to trifle with human health or life in any manner he chooses. There are laws in existence in this State governing the practice of medicine which must be adhered to by every person entering this field, and when these are violated, it must be understood that retribution will, sooner or later, overtake the offender.

There is a disposition manifest among certain young men to seek entrance into the ranks of medicine by the shortest and easiest route. Some of these have banded themselves together recently, and obtained a modification of the Medical Examiner's Law passed last year. They seem to regard this as a triumph, and to feel that they have outgeneraled some of the older and wiser members of the profession, who were interested in elevating the standard of medical education. If we mistake not, these young men have reckoned without their host, for it will be asked, by those who are competent to raise the question, of the young physicians of the classes of 1892, Have you a State License? Fancy the humiliation of the man who must answer, "No, I have not such a license; I entered the Temple of Medicine by the side door; I am an 'exempt.'"

At a time when all the countries in continental Europe, when Canada, and twenty-one of our own States, are assuming supervision of the right to practice medicine, it seems to be bad taste for any man, young or old, to array himself in opposition to the march of improvement. So, too, when a physician violates the rules of a society, he must expect to receive the discipline which pertains to such conduct, and he must stand up to such discipline in a manly way. We have nothing but kind feelings towards, and kind words for, the young men who are entering or have entered medicine, and we desire to be classed among their best friends when we counsel them, that the only way open to prosperity is through the narrow path of professional rectitude, with a proper educational basis for the true enjoyment thereof.

The Chairman of the Board of Censors deserves the thanks of the Society for the able manner in which he presented the case for expulsion, and for the indefatigable manner in which he has discharged the unpleasant duties of his office for so many years.

